# Innovative Approaches in the Synthesis and Optimization of Copper Complexes for Antitumor Therapies: A Comprehensive Review

**DOI:** 10.3390/molecules30102104

**Published:** 2025-05-09

**Authors:** Clara Maria Faria Silva, Ricardo Campos Lino, Mariana Cristina Teixeira de Moura, Anna Paula de Sá Borges, Robson José de Oliveira Júnior

**Affiliations:** 1 Laboratory of Cytogenetics, Institute of Biotechnology, Federal University of Uberlândia, Campus Umuarama, St. Piaui s/n, Uberlândia 38405-320, MG, Brazilricardo.lino@ufu.br (R.C.L.);; 2 Academic Institute of health and biological Sciencies, State University of Goiás, UnU Itumbiara, Av. Modesto de Carvalho, s/n, District Agro. Industrial, Itumbiara 75536-100, GO, Brazil

**Keywords:** anticancer, copper complexes, cytotoxic activity

## Abstract

Cancer is the second leading cause of death worldwide. Late diagnosis, low drug selectivity, high toxicity, and treatment resistance are challenges associated with pharmacological interventions. The commonly used therapies include surgery, radiotherapy, hormonal therapy, immunotherapy, and chemotherapy. Recently, Cu complexes have been studied owing to their biological functions and effects on tumor angiogenesis. In this review, we examined 23 types of cancer and revealed the use of cell lines. The synthesis of Cu complexes with ligands such as phenanthroline and thiosemicarbazones has also been reported. Such co-ligation is promising because of its high cytotoxicity and selectivity. Compared with cisplatin, Cu complexes, especially mixed complexes, showed better interactions with DNA, generating reactive oxygen species and inducing apoptosis. Nanoformulations have also been adopted to improve the pharmacological activity of compounds. They enhance the efficacy of complexes by targeting them to the tumor tissue, thereby improving their safety. Studies have also explored Cu complexes with clinically relevant pharmacophores, suggesting a “hybrid chemotherapy” against resistant tumors. Overall, Cu complexes have demonstrated therapeutic versatility, antitumor efficacy, and reduced adverse effects, showing great potential as alternatives to conventional chemotherapy and justifying future clinical investigations to validate their use.

## 1. Introduction

According to the World Health Organization (WHO), cancer is the second leading cause of death worldwide, second only to cardiovascular diseases [1]. In 2022, there were approximately 20 million new cases and 9.7 million deaths from cancers, including non-melanoma skin cancers (NMSCs). Estimates suggest that approximately 1 in 5 men or women develop cancer in their lifetime, while approximately 1 in 9 men and 1 in 12 women die from it [2].

High mortality is related to factors such as late diagnosis and factors related to the drugs used, such as reduced cytotoxic selectivity, high toxicity, and acquired tumor chemoresistance, which leads to low adherence and high evasion of treatment [3,4]. The primary antitumor treatment options include surgery, radiotherapy, hormone therapy, immunotherapy, and chemotherapy. On the other hand, emerging therapies have been showing good results, such as hormonal therapy, molecular targeting, genetics, cellular, biological, photodynamic, proton and nanomedicine.

Much research has been conducted using complexes based on metal ions in chemotherapy because the vital activities of cells and enzymes are organized by inherently existing metals [5]. Among these ions, Cu has been gaining attention in cancer research because it is an essential trace element for mammals and a component of enzymes and proteins, where it plays an important role in a range of biological processes. In addition, Cu plays an essential role in tumor angiogenesis, which is fundamental to tumor growth and metastasis, as demonstrated by the high concentration of Cu in tumor tissues [6].

Cu is an element sensitive to oxygen concentration and forms a rich variety of complexes with oxidation states +1 and +2, with a varied number of coordination and geometries [7,8]. In addition, it also has the properties of ligands, which allows the design of new metal complexes coordinated by Cu in the search for better antitumor drugs. The appropriate choice of ligands is the fundamental step in planning new syntheses. Tetrahedral geometry dominates in Cu(I) complexes, while square planar, trigonal bipyramidal, and octahedral arrangements are more frequently observed in the crystal structure of Cu(II) complexes [9].

Cu complexes have emerged over the years as an attractive chemotype for the treatment of human cancer. Several studies have shown that these complexes exhibit greater cytotoxicity, selectivity, and an ability to interfere with cell growth differently in normal and tumor cells, and they are even safer when compared to platinum-based complexes [10,11,12,13,14,15,16,17,18,19].

Therefore, the study of different types of cancer and their respective experimental methodologies has been of great relevance to the advancement of clinical science [12]. This study aimed to identify the main strategies used to develop and improve Cu complexes with antitumor activity.

## 2. Methodology

A comprehensive literature search was performed using PubMed and ScienceDirect. The keywords were applied in several different combinations such as “copper compounds” AND “antineoplastic agents” AND “pharmacological activity” NOT “clinical trial” NOT “review” in PubMed. A search was also performed using the descriptors “copper compounds” AND “cancer” in the ScienceDirect database.

The inclusion criteria for this review focused on past studies using Cu complexes as the metal center and involving biological analyses of tumor cells in vitro and/or in vivo, published between October 2019 and September 2024. Studies that tested compounds for diseases other than neoplasms, clinical studies, and literature reviews were excluded.

A total of 456 articles were identified, including 262 from the PubMed database and 194 from ScienceDirect. After reading the titles and abstracts, 280 were excluded, and 177 were selected (116 from PubMed and 61 from Science Direct). An important fact that was observed is that six articles were present in both databases, and 171 articles were counted for the subsequent analysis. The following data were collected: chemical formula of the Cu complex, type of cancer, cell line, strategies for improving the efficacy of Cu complexes (ligands, co-ligands, and formulations), type of tests (in vivo, in vitro and in silico), mechanistic action of the compounds, and efficacy and safety of the compounds in relation to cisplatin. Furthermore, all figures were created using ChemDraw software (version 12.0).

## 3. Types of Cancer and Main Cell Lines Studied to Verify the Antitumor Action of Cu Complexes

This review analyzed 23 different types of cancers, reflecting the diversity of neoplasms affecting the global population. Breast, colon, and lung cancers were the most studied types (Table 1). These findings are consistent with the high prevalence and mortality rates associated with these diseases. The widespread attention given to these neoplasms can be explained by their high incidence, which ranges from 34 to 212 times higher in countries in transition [1] as well as the ongoing search for more effective treatments and early diagnosis methods.

Breast cancer, for example, is the most common type of cancer among women worldwide and is one of the most studied [173]. Its molecular heterogeneity and different responses to treatment justify the large number of studies. Similarly, colon and lung cancers are frequently studied owing to their high mortality and the complexity of treatment [174], which calls for continuous research into new therapeutic compounds and prevention approaches. As shown in Table 1, the distribution of the types of cancer reinforces this tendency to concentrate research efforts on a few specific types of cancer.

Other types of cancer are also relevant, but there are few studies that have conducted tests with such cell types. This indicates a possible gap in the analyses conducted, as they may have specific biological characteristics and require greater scientific attention. To advance scientific knowledge [175] and search for more effective Cu metallocomplex therapies for the treatment of neoplasms, it is important to highlight the need to expand studies to other types of cancer, which are equally devastating.

In addition, in the 171 articles analyzed in this review, 108 cell lines were studied and used to investigate the effect of Cu compounds. In several studies, more than one cell line was used (Table 1).

The most frequently used strains are MCF-7 and MDA-MB-231, both derived from breast tumors. The predominance of these strains can be explained by the high prevalence of breast cancer, as well as the heterogeneity of the molecular subtypes of this cancer [176]. This makes these cells relevant models for investigating the therapeutic potential of new compounds. MCF-7 cells are often used to study the estrogen-dependent forms of breast cancer [177]. In contrast, MDA-MB-231 cells are a model of triple-negative breast cancer that tends to be more aggressive and difficult to treat [178].

The A549 strain, derived from lung cancer [179], also appears frequently, in line with the high incidence of lung cancer worldwide, with approximately 2 million new cases and 1.76 million deaths per year [180]. The HeLa strain, which comes from cervical cancer, is another widely used model because of its robustness and replication capacity, and it is a reference in cancer studies [181]. In addition, this was the first established immortal human cell line [182].

The PC-3 strain, derived from prostate cancer [183] is often used because of the increasing prevalence of this neoplasm, especially in older men [184]. The investigation of Cu compounds in this lineage is particularly important, given that prostate cancer is proving resistant to conventional treatments [185] and new therapeutic alternatives are necessary.

The HepG2 strain, originating from hepatocellular carcinoma [186], also features prominently, as liver cancer is one of the leading causes of cancer-related deaths worldwide [187]. HepG2 cells are a relevant model for evaluating the impact of therapeutic compounds on liver tumors [178]. This will enable a better understanding of the mechanisms involved in liver cancer and the effects of Cu compounds on these cells.

These cell lines allow for a controlled investigation of the effects of Cu compounds, with comparable results between different studies. Therefore, the results of this review offer valuable insights into the predominant use of cell lines as study models for exploring the therapeutic potential of Cu compounds in different biological contexts.

## 4. Strategies Used to Improve the Antitumor Efficacy of Cu Complexes (Ligand, Linker and Pharmacophore)

**Figure 1:**
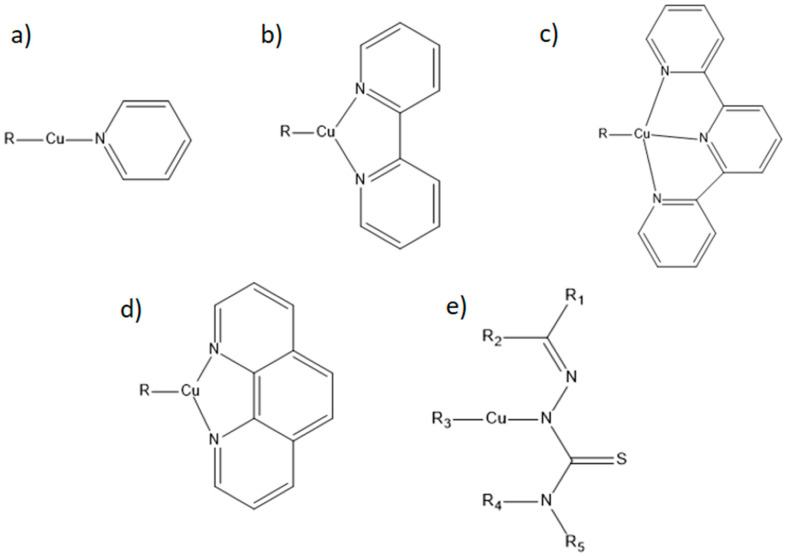
Chemical structure of the main copper-coupled ligands. (**a**)—pyridine (PYR), (**b**)—bipyridine (BYP), (**c**)—terpyridine (TERP), (**d**)—phenanthroline (PHEN), and (**e**)—thiosemicarbazone (TSC). Source: Own authorship.

In this review, the commonly used ligands in Cu complexes were phenanthroline (PHEN), followed by pyridines (PYR), bipyridines (BYP), terpyridines (TERP), and thiosemicarbazones (TSC) (Figure 1).

The PHEN (Figure 1d) ligand and its substituted derivatives, in both metal-free and coordinated states, disrupt the functioning of a wide variety of biological systems. Cu complexes are more active in the presence of heterocyclic ligands because they exhibit potent antitumor activity against various cancer cell lines through the generation of reactive oxygen species (ROS), which results in the induction of apoptosis [112].

Various studies have been conducted to improve the effectiveness of Cu-PHENs by adding binding agents. Mixed Cu compounds have attracted great interest owing to their ability to interact with DNA and their consequent nuclease actions [140]. In the present review, it was possible to observe the use of mixed Cu-PHEN as a pharmacological strategy to improve antitumor activity [20,21,57,58,120,121,136,163,167,171,188].

It is worth noting that the binding agents generally comprise molecules with biological characteristics (antioxidant, anti-inflammatory, and antitumor activities) [57]. The main mechanisms of the antitumor action of Cu-PHEN as a single ligand or mixed ligand identified in this review are described in Table 2. 

Another common ligand in Cu complexation is the heterocyclic compounds BYP and TERP (Figure 1b,c). These two compounds have characteristic optical and electrochemical properties, such as the ability to form complexes with metals. The BYP ligand has been tested as a simple ligand [148] and in combination with other ligands [21,136,140,164,167,171].

Several studies on the Cu-BYP complex have also evaluated the Cu-PHEN complex [20,21,59,140,164,171,188]. In general, Cu-BYP mixed ligands show excellent cytotoxic activity, with IC_50_ values close to 10 µM. However, when compared to Cu-PHEN mixed ligands, the latter showed greater cytotoxic activity [20,59,140,167,171,188].

Only one study has shown greater specificity of the Cu-BYP/phenylmethyltriazole mixed heterocycle ligands in the B16F10 cell line compared to that of the Cu-PHEN/phenylmethyltriazole [164]. Another study showed that the Cu-BYP/Schiff base mixed ligands were more potent in the gliobastoma cell lines U87 MG and U373 MG, while the Cu-PHEN/Schiff base mixed ligands were more potent in the neuroblastoma cell line Sh-SY5Y [167].

Regarding the TERP ligand (Figure 1c), of the seven studies identified in this review, Cu-TERP complexes have been shown to be highly cytotoxic [60,61,62], to demonstrate good selectivity [62,63], and to have better anticancer effects than cisplatin [60,62,64]. Notably, only one study [120] added a linker (PHEN, BYP, or methylimidazole), all of which were found to be more cytotoxic and selective than cisplatin. These data revealed that Cu-TERP complexes are promising antitumor agents.

Another prevalent ligand in this review is thiosemicarbazides, which comprise a group of sulfur derivatives of semicarbazones obtained by the condensation of appropriate aldehydes or ketones and thiosemicarbazides in an acidic environment. The structure of thiosemicarbazides has a significant influence on their biological activity [165] and has therefore been used as ligands in metal complexes.

Recently, TSC (Figure 1e) has been studied for their promising anticarcinogenic properties. These ligands and their transition metal complexes are biologically active compounds and anticancer agents with versatile structural properties [65]. Metal complexes with this ligand may have different cellular targets than cisplatin, raising the prospect of increasing the spectrum of action and obtaining better selectivity [10].

Fourteen articles used TSCas as ligand. In four of these articles, the results showed greater cytotoxic effects than those of cisplatin [10,66,107,149]. Two articles showed a good selectivity index [11,22], and two that carried out in vivo studies observed improved safety [10,11].

Different studies report varying mechanisms of action for Cu-TSC complexes (Table 2). Four studies used salicylaldehyde group [10,11,100,107]. In these studies, three distinct mechanistic actions were identified: inhibition of disulfide isomerase [10,11], activation of caspases 3 and 7 [100], and inhibition of organic cation transporters [107].

As shown in Table 2, the pharmacological actions of the Cu complexes differed between studies. This difference may be related to the different actions of the ligands complexed with Cu and the experimental design of the studies, which differ in terms of the methods used to investigate the pharmacological actions of the compounds.

Some past studies have presented ligand-improvement techniques to improve the binding of Cu metal complexes to DNA and increase their cytotoxicity in cancer cells. Romo et al. [23] used the ligand 1,3-bis(1,10-phenanthrolin-2-yloxy)-*N*-(4-(methylthio)benzylidene)propan-2-amine. According to the authors, the distorted trigonal-bipyramidal geometry of the Cu complex with this ligand allowed PHEN fragments to be easily accommodated in the DNA double helix. In addition, the aromaticity of these fragments improves their local hydrophobicity, thereby increasing their affinity for the hydrophobic domains of DNA. The Cu complex showed superior cytotoxicity in the cell lines studied when compared with cisplatin.

The tetramethylphenanthroline ligand is a virtually flat molecule that intercalates DNA and is highly cytotoxic. Alvarez et al. [24] evaluated the cytotoxicity of Cu complexes in various cancer cell lines. They found that Cu complexes with the L-dipeptide ligand tetramethylphenanthroline were highly cytotoxic compared to Cu complexes with the L-dipeptide ligands PHEN and cisplatin.

Another ligand, bathophenanthroline (BPHEN), has shown promising results. Du et al. [136] and Trávníček et al. [121] compared this ligand to the PHEN ligand and showed inferior activity. By contrast, Cu-L-dipeptide complexes and BPHEN showed superior activity when compared with that of Cu-L-dipeptide complexes, PHEN, and cisplatin [25]. Therefore, these ligands are potential candidates for studying in vivo activity in the treatment of aggressive tumors for which there is no curative pharmacological treatment. In addition, the Cu-naringenin/BPHEN ternary complex showed greater selectivity against cancer cells than the Cu-naringenin complex. Cell death was found to be related to the generation of ROS, loss of mitochondrial membrane potential, depletion of GSH, and GSH/GSSG ratio [122].

As can be seen, in addition to modifications to the structure of the ligand, peptide additions have been used to improve the activity of the compounds and are intended to cover a range of different side chains and increase lipophilicity. The results indicated that the use of L-dipeptides increased cytotoxic activity compared to other Cu complexes [24,25].

In the search for more effective anticancer drugs aimed at specific molecular targets, the use of a “hybrid” medicinal chemistry approach, which exploits the unique behavior of transition metal complexes conjugated to pharmacophores, can provide an advance in therapy. In the present review, the main pharmacological classes and their representatives found were selective estrogen receptor modulators (tamoxifen) [26], sex hormones and steroids (estradiol) [67], antibiotics (pefloxacin, ciprofloxacin, nalidixic acid, doxycycline, tetracycline) [12,68,101,160], anti-inflammatory drugs (indomethacin) [141], antiparasitics (albendazole) [69], and disulfiram [123].

Regarding the tamoxifen-derived ligand (TAML), Cu-TAML retained its estrogen receptor-binding activity. Its pharmacological action is related to redox imbalance, characterized by a reduction in the total thiol content and an increase in ROS production. In addition, it promotes mitochondrial swelling and the release of pro-apoptotic factors, probably due to extensive oxidative damage. The compound exhibited cytotoxic activity toward estrogen-sensitive and -insensitive breast cancer cells and overcame cancer resistance [26].

Barrett et al. [67] in their study complexed Cu-PHEN with estradiol. These complexes showed strong in vitro intercalatory interactions with nuclear DNA, ROS production, and DNA cleavage. This series of compounds showed reduced and submicromolar IC_50_ values and cellular uptake followed the order of lipophilicity, indicating that internalization occurred mainly by passive diffusion.

Several studies have investigated Cu-complexed fluoroquinolones as antibiotic-derived ligands. The wide applications of Cu ions and fluoroquinolones, such as antimicrobial, anti-inflammatory, and antiviral activities, have stimulated research into mixed Cu-ciprofloxacin/isatin and Cu-pefloxacin/isatin complexes. The antitumoral actions of these compounds are as follows: antioxidant action, inhibition of clonogenic capacity, and activation of apoptotic pathways in HCT116 cells [12,101].

Another complex synthesized using antibiotics is hydrazone/nalidixic acid. Notably, nalidixic acid has shown promising antimicrobial and antitumor activity. The complexes showed prominent methanuclease activity with cytotoxic activity against U251, UACC-62, MCF-7, and HT-29 cells. These findings stimulated new studies on this mixed compound [68].

Another synthesized metallopharmaceutical is a Cu ternary complex with doxycycline and phenanthroline (Cu-Dox-PHEN), which is highly cytotoxic and has great potential for DNA cleavage. The mechanistic actions include the generation of free radicals, ROS generation, intercalation, and DNA cleavage. The compound exhibited moderate genotoxicity, selective inhibition of B16F10 murine melanoma tumor cell growth, in vivo chemotherapeutic potential against S180 and Ehrlich sarcoma tumors [160].

Ligands derived from anti-inflammatory drugs can improve the action of antitumor compounds by reducing inflammation at the tumor site. In a study by Godínez-Loyola et al. [141] the action of the Cu-indomethacin/diimines/Schiff base complex was associated with tumor reduction due to cyclooxygenase inhibition. The release of the compound exhibited a burst effect in acidic media, which is characteristic of the tumor site.

Owing to its antiparasitic effects and recent evidence of its antitumor activity, albendazole has been used in the synthesis of coordination compounds. Cu-albenzazole complexes show cytotoxic activity against HeLa, MCF-7, PC3, and HCT-15 cell lines. Although the mechanism of action has not yet been elucidated, these findings indicated cell death by apoptosis [69].

Another drug with potential antitumoral activity is disulfiram, whose activity is enhanced in the presence of Cu ions. The Cu-Disulfiram complex easily crossed the membrane of A549 cells and accumulated intracellularly. This process triggered cell morphological changes, increased ROS, cell cycle arrest in the G0/G1 phase and apoptosis [77,123].

## 5. Mechanistic Actions of Cu Complexes

Of the 171 articles reviewed, 18 (10.5%) did not present synthesis studies in conjunction with biological studies, and 129 (75%) investigated the classic mechanisms of action of Cu complexes that are already well established in the scientific community, including the ability to cleave DNA by releasing reactive oxygen species, causing cell cycle arrest and consequently apoptosis.

Of these biological tests, 90 studies carried out cytotoxicity, DNA interaction, and cleavage tests, as well as flow cytometry marker tests to elucidate their mechanisms of action. Of these, 39 showed only studies on cytotoxic capacity, DNA interactions, and cleavage capacity. Of these, 4 presented data exclusively on DNA cleavage, while another 5 carried out only interaction tests with macromolecules and/or in silico simulations. Another 4 presented only cytotoxicity studies, and another 26 presented cytotoxicity, cleavage, or docking studies. Of the total number of selected articles, 38 did not present mechanistic studies and focused only on synthesis and proliferation tests. Another five studies conducted phototoxicity tests and in vivo experiments.

Of all the studies selected for this review, 72 (41.9%) studied other mechanisms of action and other potential targets for the antitumor activity of Cu complexes or investigated the expression of enzymes that corroborate the classic mechanism of programmed cell death.

Carcelli et al. [10] and Pellei et al. [118] proposed a new target, disulfide isomerase proteins (DPIs), which catalyzes the reduction in disulfide bonds and the oxidation of thiols. DPIs are abundant oxidoreductases that reside in the endoplasmic reticulum (ER) and play crucial roles in protein folding. However, because of the major interference between the ER and mitochondria in redox homeostasis, the inhibition of IPRs has been shown to strongly affect mitochondrial pathophysiological stability [11].

In addition, studies have evaluated the levels of reduced thiols in cells treated with metal complexes. These results supported the hypothesis that Cu(I) derivatives linked to pyrazoyl or Cu(II) linked to TSC can effectively target DPIs in colon cancer cells. This causes an imbalance in the cellular redox homeostasis, shifting it to a reduced state. In addition, morphological analysis revealed that both complexes induced a slight increase in mitochondrial size, reduced the electronic density of the inner membrane and matrix regions, and altered cristae characteristics [10,118].

Compounds that inhibit mitochondria-resident IPRs induce non-oxidative stress-mediated cancer cell death. In addition, IPRs have a binding affinity for Cu and they have been shown to play an essential role in regulating the intracellular redox state of Cu ions, which catalyzes the formation of disulfide bonds [189].

Another mechanistic action is the externalization of phosphatidylserine and the activation of endogenous caspases, both via extrinsic and intrinsic pathways, resulting in apoptosis [189]. Parsa et al. [161], Reheman et al. [142], and Vitomirov et al. [71] demonstrated that activation of caspase-3, caspase-8 and caspase-9 induced apoptosis in HeLa cells with the Cu-PHEN compound, in HeLa cells with the Cu-pyrazolone compound and in leukemia cells with the Cu-TSC compound, respectively. Two of these past studies also showed an increase in BAX protein expressions in relation to BCL-2 protein transcripts, which are pro-apoptotic and antiapoptotic regulators [142,161]. Changes in the expression of these proteins may be associated with increased cancer risk and treatment resistance.

Additionally, Reheman et al. [142] demonstrated cleavage of poly (ADP-ribose) polymerase (PARP) protein. PARP is a polymerase that participates in the DNA repair process, and its inhibition is an efficient approach for treating various types of cancers. The success of this approach has led to the approval of four different PARP inhibitors for the treatment of various types of cancer; seven different compounds are currently under clinical investigation for various indications [190].

Furthermore, Reheman et al. [142] observed three pathways, namely: PI3K/AKT, P38/MAPK, and JNK/MAPK. The study proves that the copper complex induced apoptosis by blocking the cell cycle in the S phase. Furthermore, the compound in question promoted the activation of caspase-3 and caspase-9 in HeLa cells. It inhibited the PI3K/AKT pathway and activated the P38/MAPK and JNK/MAPK pathways. It also inhibited the phosphorylation of Iκ-Bα in the NF-κB pathway activated by TNF-α, thus restricting the routine of HeLa cells.

The MAPK signaling pathway is involved in the regulation of biological mechanisms such as cell proliferation and apoptosis. In the PI3K/AKT signaling pathway, the balance between cell proliferation and apoptosis can be modulated by regulating AKT expression to inhibit tumor cell growth. The P38/MAPK pathway has been associated with the induction of apoptosis through various cellular stress signals, such as TNF-α, interleukin-1, ultraviolet radiation, hyperosmotic stress and chemotherapy, while activation of the JNK pathway can increase caspase-3 activity, which culminates in apoptosis [191,192].

Zhang et al. [124] studied Cu(II) complexes with 5-pyridin-2-yl-[1,3]dioxolo[4,5-g]isoquinoline derivatives and monitored the expression of caspase-3 and caspase-9 in seven compounds by flow cytometry, two of which showed an increase in the expression of caspases-3 and caspase-9, causing apoptosis via the intrinsic pathway.

Mutlu Gençkal et al. [185], when investigating copper compounds and other transition metals, linked to quercetin and diimines, carried out tests to evaluate the behavior of caspases-3 and caspase-7. Strong activation of this pathway has been observed in human oral squamous cell carcinoma cells (KBv200), demonstrating the induction of apoptosis via caspases-3 and caspase-7 in the Cu-PHEN/quercetin complex. This same complex showed an increase in the level of the M30 antigen, which was detected in an ELISA test for cytokeratin-18 breakdown, which refers to the action of caspases at a specific aspartate 396 (Asp396) cleavage site. This is an important indicator of apoptosis [193]. 

Another mechanism that has been studied is the inhibition of proteasomal activity. Modified cells that become tumors positively modulate the ubiquitin-proteasome system (UPS) and use it to degrade tumor suppressor proteins and prevent apoptosis [27]. Balsa et al. [82] showed that the Cu-acylhydrazone complex inhibited proteasomal activity by 30% in MCF7 cells and 47% in MDA-MB-231 cells, which helped prevent the antiapoptotic mechanism developed by tumor cells. In addition, the complex was shown to bind to the 20S proteasome activation site via molecular docking.

With regards to miRNA, they comprise a class of small non-coding RNA molecules that can silence up to 60% of coding genes. They can act as tumor suppressors or oncogenes (oncomiR). Studies have shown that oncomiR suppression therapy or increased expression of tumor suppressor genes is effective in treating cancer. Overexpression of miR-21 and miR-155 is associated with apoptosis, proliferation, and invasive potential via silencing of programmed cell death 4 (PDCD4) and liver kinase B1 (LKB1), respectively [194].

miR-206 and miR-133b are members of the miR-bicistronic cluster. Higher levels of miR-206 increase apoptosis and prevent cancer formation, while miR-133b is an oncomiR that activates the ERK and AKT1 pathways, which are important for oncogenesis [114]. Measurement of miRNA levels via RT-PCR represents an additional level of investigation into the antitumorigenic effects of the selected compounds. Petronijević et al. [195] analyzed the RNA of cells treated with three Cu-quinoxalinone complexes. Among them, miR-21, which has high oncogenic potential, showed no changes. By contrast, two compounds increased the levels of miR-206, and all three increased the levels of miR-155. These data show that the compounds exert better anticancer activity by stimulating suppressor genes and inhibiting tumor-promoting genes.

Tubulin is one of the main targets in cancer treatment because it plays essential roles in cell division and intracellular transport. Inhibiting the formation of microtubules induces cell death by apoptosis, making them a new target for chemotherapy studies aimed at inhibiting the polymerization or depolymerization of microtubules [70].

Tubulin disassembly experiments showed that the two Cu-benzazozine complexes are effective microtubule-destabilizing agents that bind to the colchicine site, which was also confirmed by molecular modeling tests [28]. The binding of a metal complex to the tubulin-colchicine pocket is unprecedented. Hossan et al. [196] treated cells for 24 h with a semi-synthetic Cu-cardamonin compound, stained them with DRAQ5 and anti-α-tubulin monoclonal antibody. and investigated them morphologically using confocal microscopy. The images revealed the induction of a microtubule-disrupting agent (MDA) in cancer cells, such as multinucleation, nuclear fragmentation, and disruption of the microtubule network.

Topoisomerases are proteins that are essential for cellular processes such as replication, transcription, DNA duplication, chromatin assembly, and chromosome segregation, and are capables of modifying the topological properties of DNA. For example, they regulate the level of supercoiling in double helices. These inhibitors act mainly through interactions with topoisomerase I and tumor DNA, thus inhibiting the replication of tumor cells [108].

Wittmann et al. [109] studied complexes of Cu-latonduines and Cu-quinolines, which proved to be DNA-intercalating agents and subsequently caused poisoning of topoisomerases I and II, which is considered a mechanism of action of quinolines. By contrast, Pósa et al. [152] demonstrated the inhibition of topoisomerase by Cu-TSC complexes using electrophoresis. This process leads to cell cycle blockage in many tumors cell lines and consequently generates numerous DNA breaks during non-homologous recombination. The result of this is lethal chromosomal damage, such as breaks, which subsequently lead to nonhomologous recombination, and the accumulation of errors in the genome causes cell apoptosis.

## 6. Nanoformulations as a Strategy to Improve the Effectiveness and Safety of Cu Complexes Used as Antitumor Agents

The use of nanoformulations is a therapeutic strategy that uses nanoparticles to transport drugs or other active ingredients, thereby improving quality, safety, and efficacy. In the present review, six studies used the nanoencapsulation of Cu compounds as an improved strategy to reach the tumor environment more effectively and safely.

Encapsulation involves the modification of different physicochemical and biochemical characteristics, including solubility, stability, and rapid release. This method prevents drug degradation, increases therapeutic efficacy, and alsos reduces side effects. Nanoparticles can increase the permeability and retention effect, so they are able to increase the concentration of the drug inside the tumor, controlling drug release and specific targeting [141,197]. These characteristics give nanocomplexes an important role in cancer therapy and make them promising candidates for replacing conventional chemotherapy.

This was observed in a study using Casiopein^®^, CasIII-ia nanoencapsulated in niosomes designed and optimized with Quality by Design (QbD) tools for intravenous (IV) administration. In vivo analyses showed good efficiency and lower toxicity compared with the compound and free cisplatin. The MDA-MB-231 strain showed greater CasIII-ia activity in the period of up to 48 h absorbed by a diffusion process, demonstrating better distribution of the drug and low toxicity [37].

In a combination of Cu and cetyltrimethylammonium bromide (Cu-CTAB) in gallium oxide nanoparticles (Cu-CTAB + GaONPs), the formulation showed an IC_50_ of 0.2 µg/mL in hepatocellular carcinoma cells (HepG-2). This showed greater antiproliferative power when compared to Cu-CTAB and GaO-NPs administered alone. In Wistar rats, the recovery of damaged tissue was observed histologically, and biochemical tests showed a reduction in liver function markers alanine aminotransferase (ALT), aspartate aminotransferase (AST) and a decrease in the transcription of tumor markers such as alpha-fetoprotein (AFP), transforming growth factor beta (TGF-β1), α-L-fucosidase. Meanwhile antioxidant markers (SOD), apoptosis markers (caspase-3 mRNA) and arginase showed high values in real-time polymerase chain reaction (RT-qPCR) [158]. Gallium oxide nanoparticles can be used as multifunctional drug carriers because they easily penetrate cell membranes and, owing to their luminescent properties, facilitate their distribution within cells [198].

Pinho et al. [106] used a pH-sensitive liposome with Cu-PHEN.CL_2_. Its use in the treatment of rectal colon cancer cells maintained the cytotoxic properties of unformulated Cu-PHENs. This treatment caused a reduction in glycerol permeability and impaired cell migration, probably owing to the inhibition of aquaglyceroporins. In a syngeneic murine colon cancer model, the nanocomposite significantly reduced tumor progression compared to the control group and those treated with unformulated Cu-PHENs. No toxic side effects were observed, leading to a 50% reduction in the rectal colon cancer tumor volume. This study highlighted the maximization of biological activity using a lipid-based nanosystem [106,153].

To overcome this deficiency in the solubility of a Cu diethyldithiocarbamate (Cu-ET) complexes, the complexes were prepared with nanoparticles that were dispersed in bovine serum albumin (BSA) (Cu-ET-NPs). Such therapy generates free hydroxyl radicals and increases ROS, mediated by glutathione, which is crucial for the inhibitory role of human hepatocellular carcinoma cells (HepG2). Consequently, they induce polyubiquitination of proteins from cancer cells inoculated into mice [199].

The third generation Casiopein^®^, [Cu(N-N)(Indo)]NO_3_, was synthesized and nanoencapsulated, where Indo is deprotonated indomethacin and N-N is BYP or PHEN. The complexes showed high cytotoxicity compared to cisplatin and demonstrated anticancer performance with the characteristic and multifunctional mechanisms of action of their peers. The formulation used chitosan hydrogels, which are responsive to changes in the environment such as pH and temperature. These characteristics make them suitable as anticancer drugs, as tumor microenvironments are characterized by an acidic pH and increased temperature. The release of the compound showed a burst effect and was faster under acidic conditions in the first six hours, with a cellular absorption mechanism by passive diffusion; however, the study did not carry out in vivo tests after encapsulation [141].

The synthesis of the Cu-hydrazone nanocomplex resulted in high inhibition of HepG-2. The antitumor activity of the Cu-hydrazone nanocomplex is discussed in relation to its chemical structure, Cu (II)-reducing capacity, and potential inhibitory effect on the cyclin-dependent kinase 2 (CDK2) enzyme, as visualized by molecular docking tests [197]. A similar therapy was presented in a previous study that identified cell death via apoptosis [200].

## 7. Effectiveness and Safety of Cu Complexes Compared to Cisplatin

Cisplatin is one of the best-known and most widely used metal-based chemotherapeutics for cancer [201,202]. However, because of their high toxicity, poor tolerance, and high cost, other transition metal complexes, including Cu complexes, have been synthesized and tested as chemotherapeutic drugs [29].

In this review, 68 studies found one or more Cu compounds with a high cytotoxicity for some cell lines and with IC_50_ values lower than that for cisplatin. In 34 articles [6,9,13,14,15,16,17,18,19,22,25,65,72,73,74,75,76,82,99,101,102,103,110,122,124,136,137,142,150,159,160,171] one or more compounds showed good selectivity for tumor cells and in twelve [10,11,31,37,77,106,138,156,157,160,169,171], the compounds showed more favorable safety data than cisplatin, making these promising antitumor agents. However, some cases have shown that cisplatin proved to be more effective than the tested compound [19,58,137,145]. 

Some in vivo studies have demonstrated the greater safety of Cu complexes, Casiopein^®^ [37,169], TSC [10,11], dithiocarbazate [138], hydrazide [30,171], and triphenylphosphine [77], than cisplatin. In these studies, mice that were treated with the Cu complexes and afore mentioned ligands showed lower body weight loss than those treated with cisplatin, which can be inferred from the lower incidence of anorexia.

In addition, other safety data related to Cu complexes have been reported, such as the non-induction of mutagenicity, recombinogenicity, and carcinogenicity in *D. melanogaster* with the Cu-PHEN/hydrazide compound [156]; an improvement in the survival rate of mice with Ehrlich ascites tumors with the Cu-Dox/PHEN compound [160]; and an absence of toxic side effects with the Cu-PHEN compound [106].

Although copper compounds may exhibit lower systemic toxicity and greater selectivity compared to cisplatin, their efficacy can vary significantly depending on the tested compound and the type of cancer [62,189,203]. Such compounds can induce cell death through mechanisms distinct from cisplatin’s DNA-binding meanism. This difference may affect their efficacy and selectivity in certain types of cancer. However, it does not diminish the therapeutic potential of copper compounds, opening the possibility for further studies that will strengthen their use as a potential chemotherapeutic agent [204,205].

However, some studies have shown unsatisfactory safety data, such as signs of behavioral toxicity and mortality with Cu-thioxoimidazolone complexes [16], systemic toxicity with significant histopathological changes in the liver of mice with Cu-pyridine with halogen substitution [14], and a significant decrease in monocyte count with Cu-acylhydrazone [171].

## 8. Conclusions

In this review, we highlighted the diversity of neoplasms investigated in recent studies covering 23 types of cancer, with special attention paid to breast, colon, and lung cancers, which are the most studied. However, the limited amount of research dedicated to other types of cancer reveals a gap that demands greater scientific attention, given the potential for specific biological characteristics in these cases. Thus, among the 171 articles analyzed, there was a predominance of studies with cell lines, such as MCF-7, MDA-MB-231, A549, PC-3, HepG2, and HeLa, which served as the main models for evaluating the effects of Cu compounds and their impact in different tumor contexts.

In addition, this review has highlighted the versatility of Cu complexes, especially those with ligands such as phenanthroline (PHEN), pyridines (BYP and TERP) and thiosemicarbazones (TSC), for potential antitumor applications. The Cu-PHEN, Cu-BYP, and Cu-TERP complexes showed high cytotoxic activity, with emphasis on the mixed complexes and the use of linkers, which increased the ability to interact with DNA and the action of nucleases, thereby increasing the effectiveness and selectivity compared to cisplatin. In particular, the Cu-TSC complexes have various mechanisms of action, including the inhibition of specific enzymes and activation of apoptotic pathways. These data suggest that Cu complexes with different ligands have promising potential as therapeutic alternatives against cancer, especially for tumors that are resistant to conventional treatments.

The impact of structural modifications and the use of ligands to improve the cytotoxic activity and selectivity of compounds toward DNA and cancer cells has also been explored. Recent studies have shown that the use of ligands such as PHEN, tetramethylphenanthroline, bathophenanthroline (BPHEN), and dipeptides increases the affinity of Cu for DNA and thus improves cytotoxicity when compared to conventional treatments such as cisplatin. In addition, complexes derived from clinically applied drugs, such as tamoxifen, estradiol, fluoroquinolones, anti-inflammatories, albendazole and disulfiram, showed promising antitumor actions when combined with Cu ions, indicating a possible “hybrid” chemotherapy approach capable of tackling tumors more effectively.

A review of 171 articles revealed that most studies on Cu complexes focused on classic mechanisms of action, such as DNA cleavage, caspase activation and induction of apoptosis by reactive oxygen species (ROS). Some studies have explored new targets, such as protein disulfide isomerases (DPIs) and specific signaling pathways (PI3K/AKT, P38/MAPK, and JNK/MAPK), which are essential for redox homeostasis and apoptosis. In addition, proteasome inhibitors and miRNA modulators have emerged as potential antitumor agents, whereas the inhibition of tubulin and topoisomerases has shown efficacy in interrupting cell proliferation and inducing apoptosis. These studies showed that Cu complexes offer multiple routes of action against tumor cells.

Nanoformulation of Cu compounds are emerging as a promising approach for cancer treatment, using nanoparticles to increase effectiveness and safety by concentrating the drug in the tumor tissue and thus reducing adverse effects. Compounds such as Casiopein^®^ and combinations of Cu with other agents have shown significant antiproliferative effects, especially in types of cancer such as hepatocellular carcinoma. In comparative studies, Cu compounds have been shown to be safer than cisplatin with lower toxicity in experimental models. However, these findings reinforce the potential of Cu complexes as therapeutic agents and justify the need for future research to validate and improve their clinical use.

## Figures and Tables

**Table 1:** Cancer types and cell lines studied. Other types include urinary bladder cancer (RT-4), human tongue squamous cell carcinoma (TCA8113), thyroid carcinoma (BCPAP), extrahepatic bile duct carcinoma (TFK-1), esophageal carcinoma (Eca-109), and murine mastocytoma (P815).

Type of Cancer	Cell Lines	Total
Breast cancer	MDA-MB-231 [11,14,20,21,22,23,24,25,26,27,28,29,30,31,32,33,34,35,36,37,38,39,40,41,42,43,44,45,46,47,48,49,50,51,52,53,54,55], MMT06056 [56], MCF-7 [3,6,11,14,17,19,20,21,22,25,26,29,33,35,40,43,45,50,51,54,55,57,58,59,60,61,62,63,64,65,66,67,68,69,70,71,72,73,74,75,76,77,78,79,80,81,82,83,84,85,86,87,88,89,90,91,92,93,94,95,96,97,98], T47D [29], JC [56], ZR-75-1 [33], SUM-159 [99], BT-549 [49], HS-578 [49], and 4T1 [30,31,45].	114
Colon cancer	HCT 116 [12,13,52,63,65,77,89,100,101,102,103,104,105], CT-26 [51,61,98,106], LoVo [10,88], Colo-205 [78,80,100,104,107,108,109,110], LS-180 [111], Colo-320 [70,100,107,108,109], HT29 [39,61,68,112,113], SW620 [9,15], SW1116 [97], LS174T [52,75,112,114,115], SW480 [9,15,77,116], CaCo2 [17,112], HuTu80 [117], HCT-15 [10,11,59,67,69,86,91,118,119], T-24 [42], HCT-8 [64], and DLD-1 [30,33,40].	68
Lung cancer	A549 [6,17,19,20,21,23,24,25,40,52,54,60,62,63,64,71,73,78,80,83,87,88,90,92,97,100,114,115,116,120,121,122,123,124,125,126,127,128,129,130,131,132,133,134,135], Sk-lu-1 [59], Hop-62 [136], A-427 [137], LCLC-103 [137], H157 [119], NCI-H460 [68,110,138,139], NCIH1975 [77], NCI-H23 [139], and M109 [56].	57
Uterine cancer	HELLA [4,6,16,17,19,20,32,35,39,47,60,62,69,71,76,78,87,88,114,115,117,124,132,140,141,142,143,144,145,146], MES-SA [147], SISO [137], and ECC-1 [39].	33
Prostate cancer	PC-3 [9,10,15,17,19,20,32,39,42,43,44,47,53,59,68,69,71,80,86,92,148,149,150,151] DU-145 [39,151], LNCaP [148,150], Myc-CaP [148], and 22Rv1 [17].	28
Liver cancer	HepG2 [6,16,37,44,58,66,79,81,89,93,94,111,113,132,139,145,152,153,154], HL-7702 [124,136], SMMC7721 [155], Bel-7402 [60,72], Bel-7404 [77], H22 [156], and HCCLM3 [37,157].	30
Ovarian cancer	SK-OV-3/DDP [136,158], 2008 [10,11,56,67,118,119], A2780 [13,17,24,35,65,67,86,121,159], CH1/PA [100,116], OVCAR3 [68,149], and NCI-ADR/RES [68].	22
Leukemia	THP-1 [85,110], K562 [29,49,59,68,86,128,160,161,162], KG1a [161], HEL [49], Jurkat [29,85,135,163], L1210FR [56], HL60 [49], and Molt-4 [110,138].	21
Human melanoma	B16-F10 [83,113,147,160,164], A375 [10,19,33,46,65,71,92,111,165], G361 [165], MV3 [43], SK-MEL-28 [165], and UACC62 [55,68].	19
Glioblastoma glioma	A172 [32,47], LN229 [32,33,35,47,166], U-87 [32,47,53,167], U-251 [59,63,68] U373-MG [167], T98G [111], and C6 [68,168].	19
Pancreatic cancer	PANC-1 [17,28,63,73,169], PaCa-2 [170], DAN-G [137], Capan-2 [132], BxPc-3 [10,92,107,169], ASPC-1 [169], and PSN-1 [10,11,118].	16
Neuroblastoma	SH-SY5Y [139,167] and IMR-32 [78,79,91,145].	6
Osteosarcoma	HOS [17,121], MG-63 [21,23,171], and 143B [170].	6
Squamous cell carcinoma	A431 [11,67,118,119,172].	5
Gastric cancer	MGC803 [77,124,132] and BGC823 [155].	4
Renal cancer	RD0995 [56], TK10 [55], RENCA [56], and 786-0 [68].	4
Lymphoma	Ragi [135], DL [18], and U937 [29,110].	4
Others	RT-4 [137], TCA8113 [152], BCPAP [10], TFK-1 [155], Eca-109 [60], and P815 [56].	6
	Total strains studied	110

**Table 2:** Main ligands and co-ligands of the Cu complex and the pharmacological action.

Single Ligand Pharmacological Action	Pharmacological Action of Complexes with Ligands
PHEN: Delayed apoptosis and release of interleukin 6 (IL-6) [23]; inhibition of aquaglyceroporins, which reduced glycerol permeation and impaired cell migration [106]; induction of ROS and apoptosis [112]; effective binding with antiapoptotic proteins of the BCL family [76]; not investigated [147]. BYP: Cell cycle arrest in the G0/G1 phase and inhibited signaling pathways regulated by Cathepsin D [148]. TERP: ROS production, inhibition in the G0/G1 phase and subsequent apoptosis [63]; ROS production and apoptosis [61]; strong affinity of the compounds to bind to DNA as intercalators and induce DNA conformational transitions [60]; not demonstrated [64]. TSC: inhibition of protein disulfide isomerase [10,11]; inhibition of EGFR protein [66]; activation of caspases 3 and 7 leading to apoptosis [100]; G2/M phase cell cycle arrest and DNA damage [165]; inhibition of OCT1-3 [107]; accumulation of cells in the sub-G1 fraction, as well as reversible arrest in the G0/G1 and G2/M phases in K562 and KG1a cells [161], externalization of phosphatidylserine and activation of caspase-3 leading to apoptosis, increased formation of ROS in K562 and KG1a cells [161]; reduction in catalase activity [25]; inhibition of glutathione synthesis [25,152]; inhibition of topoisomerase [152]; not demonstrated [3,19,65,129].	PHEN + Schiff base: ROS [167]; DNA and FGRF receptor binding; increased planarity, chelation [58]. PHEN + diplacone: G2/M cell cycle arrest; ROS [121]. PHEN + biotin: DIP inhibition together with nuclear DNA and apoptosis [52]. PHEN + phenylmethyltriazolol: Methanuclease activity and DNA degradation [164]. PHEN + hydrazide: Reduced expression of the Ki-67 substance and the Cyclin D1 protein [156]. PHEN + hydroxyphenylimino: Apoptosis induced by intrinsic and extrinsic pathways [57]. PHEN + 8-hydroxyquinolone: Apoptosis via mitophagy and ATP depletion [136]. PHEN + hydrazone: Apoptosis and DNA cleavage [21,171]. PHEN + quercetin: Apoptosis; caspase 3/7 activity; mitochondrial depolarization [185]; PHEN + hydrocyananthracene: inhibition of topoisomerase I, ROS, DNA cleavage [188]; PHEN + phenolate; PHEN + naphthenolate: π-stacking interaction; DNA cleavage [140]. PHEN + TERP: Not demonstrated [120]. BYP + 8-hydroxyquinoline: Apoptosis via mitophagy and ATP depletion [136]. BYP + Schiff base: ROS [167] BYP + phenylmethyltriazolol: Elimination of superoxide; methanuclease activity [164]. BYP + quercetin: Apoptosis; caspase 3/7 activity; mitochondrial depolarization [185]. BYP + hydrazone: apoptosis and DNA cleavage [21,171]. BYP + hydrocyananthracene: Inhibition of topoisomerase I, ROS, DNA cleavage [188]. BYP + phenolate; BYP + naphthenolate: π-stacking interaction; DNA cleavage [140]. TERP + phosphine: Not demonstrated [62]; TERP + BYP: Not demonstrated [120]. TSC + phosphane: Apoptosis [149].

PHEN: phenanthroline; BYP: bipyridine; TERP: terpyridine; PYR: pyridine; ROS: reactive oxygen species; DNA: deoxyribonucleic acid; TSC: thiosemicarbazones; EGFR: epidermal growth factor inhibitor; OCT: organic cation transporter.
